# Domestic

**Published:** 1841-08-14

**Authors:** 


### MEDICAL EXAMINER.

DEVOTED TO MEDICINE, SURGERY, AND THE COLLATERAL SCIENCES.

No. 33.] PHILADELPHIA, SATURDAY, AUGUST 14, 1841. [Vol. IV.

### DOMESTIC.

*New York Medical Gazette.*—A new journal, under this title, has taken the field in New York. The quarterly journal, so ably conducted in that city during the last two years, has been suffered to expire, and a weekly journal is now started, under new auspices. We welcome, with pleasure, this addition to the ranks, which promises to be a useful and agreeable one. The Gazette is edited by Wm. C. Roberts, M. D.

---

*Case of Procidentia Uteri, successfully treated by pressure with adhesive straps to the Tumour.* By J. Kearny Rodgers, M. D., Surgeon to the New York Hospital, &c. &c.—Margaret Murphy, born in Ireland, aged 30 years, was admitted under my care into the New York Hospital, June 18th, 1840, with procidentia uteri.

She was the mother of five children, and her last was born about two years ago. About twelve months since, she had pain in the back with slight descent of the uterus, on remaining any length of time in the erect posture, or after long walks. She was accustomed to very hard work, and in the habit daily of carrying very heavily laden baskets on her arm, which she continued to do until about three months since. At that time the os tincæ began to protrude from the vulva, and continued daily to descend more and more.

About a month since, the uterus having entirely protruded, the friction of her clothes caused the ulceration of the mucous membrane of the everted vagina, near the os uteri; but as she continued her attention to her household duties, the tumour enlarged still further, and did not return within the external parts on her assuming the horizontal posture.

For the week preceding her admission, she was unable to attend to her avocations, and for four days had severe pains in the back and loins, and bearing down pains like those of labour.

On her admission, the uterus and bladder, covered by everted vagina, formed a pear-shaped tumour between the thighs, about five inches in length and three in breadth, very similar to the plate (No. 1) in the first volume of "Clarke on the Diseases of Females atttended by discharges." Around the os uteri, the mucous membrane was ulcerated for the space of an inch, and the remainder had acquired almost the firmness and appearance of common integument.

The meatus urinarius was seen in the upper part of the tumour, and a catheter introduced in it, and passed downward and back, drew off the urine.

The tumour could not by pressure be passed within the labia.

The patient was ordered to observe the horizontal posture, and the bowels were relieved by enemata.

Having recently seen very beneficial effects from the constant and uniform application of firm pressure by adhesive straps in hydrocele, I determined to adopt the plan in this case, and the dressings were well applied by the house surgeon, Dr. MacNevin, Jr. The pressure was made from below upwards, and the straps placed rather obliquely, each overlapping the other.

She had now frequent desire to pass water, and it was accompanied with so much pain that she was relieved by the catheter.

After the application of the straps, there was a discharge of mucus from the os uteri. The tumour diminishing rapidly under the use of the straps, it became necessary to re-apply them every other day. A suspensory bandage was also applied over the straps to support the parts.

June 23.—The whole tumour disappeared within the labia during the night while she was asleep. The ulcer had begun to heal before the return of the uterus, and from this time it cicatrized rapidly.

There was now no further protrusion, and when the ulcer had healed, a large pessary was introduced to keep the uterus in situ.

The patient was discharged, *cured*, July 11, 1840.

The application of the adhesive straps to the uterus for the cure of procidentia, is, I believe, original with myself. I know of no case in which the plan has been adopted by others.—*N. Y. Med. Gaz.*

---

*Necrology.*—Died, on Monday, July 12th, Wm. James McNevin, M. D., late Professor of Chemistry in the College of Physicians and Surgeons, and Professor of Materia Medica in Rutgers' Medical School, at the advanced age of 70.—*Ibid.*

---

*Neuralgia Facialis.* By D. S. Grandin, M. D.—As the complaint which is mentioned at the head of this article is one of frequent occurrence and observation, as well as of solicitude in the practice of every dentist, and every physician, permit me to offer a few thoughts upon the treatment of this disease, in *certain cases*, as connected with my own observation and experience.

In the great majority of those cases of this affection to which I now refer, the teeth are, or appear to be involved, and a defective state of a tooth, immediately connected with the painful symptoms, is generally, though not always, found to exist; but not so deeply as to expose the nerve. As there generally appears so immediate a dependence of all the symptoms upon the supposed offending tooth, its removal is not only earnestly desired by the patient, but advised by the dentist, as the only efficacious treatment that can be adopted; particularly, as any operation upon it, with a view to its preservation, has no influence upon the tortures which seem to proceed from it. The tooth has usually the same good and healthy appearance as other teeth in cases where filling is indicated; it likewise may not be at all sensitive upon the application of the instruments for the purpose of examination, or any operation upon the defective part, and the action and pressure upon it may be attended with agreeable sensations, although pressure or concussion is likely to produce pain after the sufferings of the patient have been so protracted as to involve the periosteum.

In July, 1839, I was afflicted with a distressing neuralgia, such as I have described, which deprived me of ease by day and sleep at night. The pain seemed to proceed from the second right inferior bicuspis, shooting to the ear and over the whole side of the face to the top of the head, and causing my right eye and all my teeth to ache. I treated the case as I was accustomed to do patients similarly affected before resorting to extraction. I applied various remedies to the tooth, and lived under the influence of sulphate of morphine for a week; but no sooner had the effects of the anodyne passed away than all my sufferings returned. I called upon two of our most skilful dentists; they expressed but one opinion, and advised extraction. I should have given the same advice to any other person consulting me in a similar case after trying remedies so long as I had done. But determining to avail myself of the full benefit of all that could promise relief, without submitting to the loss of a tooth so valuable, and removing a link in a yet unbroken chain of dental structure, I continued to suffer another week without diminution. Within that time I called upon both my professional friends several times, but unfortunately did not find them at home. My sufferings had now become so intolerable that, in a paroxysm of anguish and despair, I seized the forceps and endeavoured to extract the tooth; but not possessing the same command over the instrument that I should have done had any other patient been in my hands, I broke off the crown and left the pulp exposed and projecting above the fang. I seized it with a pair of dissecting forceps and drew it out, but still experienced no alleviation of pain, and again had recourse to morphine to quiet my excited system till the next morning. The ensuing day, standing before a mirror, I examined the stump and the contiguous parts. I found that a very small process of the gum, between the broken tooth and the first molar, folded over the edge of the broken part, upon raising which all my pangs returned. It did not exceed in size the head of a common pin, but was properly, and to all intents, an irritable ulcer, and I proceeded to treat it upon the same principles that I would an irritable ulcer found upon any other part of the body of the same or greater dimensions. I immediately applied potassa to the gum, and destroyed the irritable part. The pain, though exquisite, was far more endurable than the neuralgia, and subsided in half an hour. The neuralgia immediately disappeared and has never returned. On the left side of the under jaw my first molar tooth is decayed in two places; one in the centre, the other in the anterior side. I plugged the centre myself nine years ago. The cavity in the side was plugged by my friend and instructor, Dr. S. S. Fitch, of Philadelphia, in 1834. The filling was taken out of both places three years since, to examine the tooth, and it was refilled by my brother, Wm. E. Grandin, after ascertaining that the cavities had undergone no change—and it is now in as perfect a state of preservation and usefulness as it was the day it was first filled.

In the month of October, 1840, being on a journey, I took a severe cold, one of the effects of which, was a neuralgic affection on the left side of my face, in every respect resembling the case described above. The pain appeared to proceed from the tooth last mentioned. Passing a tooth-pick between the teeth, I detected the same irritability of the interstitial process of the gum next to the decayed tooth as was discovered in the case already recorded. I touched it with caustic potash and obtained the like result. I have been consulted several times since by patients complaining in the same manner where the teeth presented the like appearance to the case first described, or had been filled as in the second; I have pursued the same plan of treatment, and have succeeded in giving them permanent relief without a single instance of failure. There can be no doubt, from the above details, that many teeth are sacrificed that might, without doubt, be preserved, and remain useful for a long period; and it is with a view to promote so desirable an object, that I have reported the above cases; for, when not myself only, but others occupying the very highest station in the profession, have erred in making a correct diagnosis in my own case, it is scarcely to be presumed that others have not generally fallen into the same error.

There is no doubt, in my own mind, that the entire extraction of the tooth first mentioned would have resulted in the cure of the neuralgia and the healing of the irritable part; and I believe the removal of the molar tooth in the last mentioned case would have cured the neuralgia; but the only solace I have for the loss of so valuable a tooth, and the destruction of the continuity of the dental arch, is the having discovered a means of preserving the teeth of others in similar cases. Care must be taken to apply only just so much of the caustic as will be sufficient to effect the necessary indication, or a deep injury may be inflicted upon the part, aggravating the already existing symptoms, and requiring a long time to heal. Perhaps the lunar caustic would be equally efficacious, but as I prefer the potash, I have not tried it.

The method I have used of applying the caustic, is to cut a splinter of wood (without regard to the kind) so as to pass it between the teeth, then touch the end of it into the potash, so as to obtain the smallest possible quantity sufficient to produce action enough to be felt, and touch the gum. The immediate and copious flow of saliva will be sufficient to prevent its burning too deep, and in a few minutes the pain subsides.

Those cases of neuralgia depending upon the exposure of the nerve of a tooth, or upon inflammation of the periosteum when the nerve is destroyed, I have not thought proper to bestow a passing notice upon, as they do not come within the range of the present inquiry.—*Am. Journ. of Dental Science*.

